# Cost and Threshold Analysis of the *FinishIt* Campaign to Prevent Youth Smoking in the United States

**DOI:** 10.3390/ijerph15081662

**Published:** 2018-08-06

**Authors:** Brian W. Weir, Jennifer Cantrell, David R. Holtgrave, Marisa S. Greenberg, Ryan D. Kennedy, Jessica M. Rath, Elizabeth C. Hair, Donna Vallone

**Affiliations:** 1 Department of Health, Behavior and Society, Johns Hopkins Bloomberg School of Public Health, Baltimore, MD 21205, USA; bweir3@jhu.edu (B.W.W.); jennifer.cantrell@nyu.edu (J.C.); rdkennedy@jhu.edu (R.D.K.); jrath@truthinitiative.org (J.M.R.); ehair@truthinitiative.org (E.C.H.); 2 Schroeder Institute at Truth Initiative, Washington, DC 20001, USA; mgreenberg@truthinitiative.org; 3 School of Public Health, University at Albany, State University of New York, Albany, NY 12144, USA; dholtgrave@albany.edu; 4 College of Global Public Health, New York University, New York, NY 10012, USA

**Keywords:** tobacco, youth, prevention, mass-media, social media, economic evaluation, cost analysis, threshold analysis

## Abstract

In 2014, Truth Initiative launched the national *FinishIt* campaign to prevent smoking initiation among youth and young adults. The significant changes in the communications landscape requires further analysis to determine resource requirements for public education campaigns relative to their impact. This analysis estimates the cost of the *FinishIt* campaign based on data from expenditure records and uses published estimates of the lifetime treatment costs and quality-adjusted life years associated with smoking. The total cost of the *FinishIt* campaign for 2014–2016 was $162 million. Under assumptions associated with the pessimistic base-case (no medical care costs saved through prevention), 917 smoking careers would need to be averted for the campaign to be cost-effective. Assuming smoking leads to increased medical care costs, 7186 smoking careers would need to be averted for the campaign to be cost-saving. Given these thresholds (917 and 7186) and the estimate of the impact of the previous truth campaign, the investments in the Truth Initiative’s *FinishIt* campaign are likely warranted for preventing smoking careers among youth and young adults.

## 1. Introduction

Funds from the milestone 1998 Master Settlement agreement between the between the state Attorneys General of 46 states, five U.S. territories, the District of Columbia and the five largest cigarette manufacturers in America were designated to establish a nonprofit organization to deliver an effective national youth anti-smoking program. As a result, Truth Initiative (formerly the American Legacy Foundation) was formed and the non-profit launched truth^®^, a national mass-media smoking prevention campaign primarily targeted at a youth audience aged 12–17 in the early 2000s. Research has since demonstrated that the campaign changed youths’ beliefs about and attitudes toward tobacco [1,2,3,4,5], prevented more than 450,000 youth from smoking over four years [4], and was cost-saving [6].

By 2013, there were substantial changes in the epidemiology of youth tobacco use. In 2013, only 9% of 12–17 year olds in the US smoked cigarettes, a decline from 23% when the truth campaign initially launched [7]. Evidence suggested that smoking initiation was increasing among young adults, and that young adults were open to experimenting with new and emerging tobacco products entering the market [8,9]. In addition to changing patterns of tobacco use, media and communications have evolved considerably with the proliferation of digital and social media. An explosion of media channels has increased consumer choice and consumption while making it more difficult to reach large consumer audiences, leading to significant audience fragmentation. Digital devices enable constant media access and multitasking. As a result, reaching youth audiences requires utilizing multiple media channels to deliver culturally-relevant campaign content to engage young audiences on the issue of tobacco use. At the same time, government agencies such as the Food and Drug Administration (FDA) and the Centers for Disease Control and Prevention (CDC) were also promoting national campaigns to reduce smoking, focusing on high-risk youth ages 11–17 and current adult smokers.

In 2014, Truth Initiative sought to reposition the truth campaign message to be highly relevant to a contemporary youth and young adult audience (aged 15–21) not already targeted by other national anti-tobacco campaigns. While most of the audience was not at risk for smoking initiation, the goal was to leverage the social power of the majority who don’t smoke to influence the minority who still do to “be the generation that ends smoking.” From 2014–2016, the truth’s *FinishIt* campaign employed an integrated marketing campaign approach, using paid media, digital engagement opportunities, and in-person experiences to connect with the target audience (Table 1). 

Given new mass communication platforms, and evolving tobacco use patterns and product categories, it is essential to examine the level of resources required for such a campaign in relation to the potential benefits to help evaluate the substantial investment. The aims of the current study are to: (1) establish the cost of the *FinishIt* campaign and (2) determine how many smoking careers would need to be prevented for the campaign to be cost-saving or cost-effective.

## 2. Methods

For both the expenditure calculations and the threshold analysis, we follow standard methods for economic evaluation in health [10,11,12]. Intervention costs (C), based on the Truth Initiative’s expenditure records, included the following: (1) development, production, and delivery of television, radio, digital, and cinema elements; (2) development, production, engagement, and analytics of social media outlets and webpages; (3) development and delivery of grass-roots summer *FinishIt* tours following youth music events throughout the U.S.; (4) formative, process, and summative evaluations and research on the campaign’s target audience; and (5) salaries of Truth Initiative staff directly contributing to the campaign (Table 2).

We used the cost estimate to calculate the number of smoking careers that would need to be averted (A) for the campaign to be cost-saving (total smoking treatment costs saved exceeding intervention costs) or cost-effective (net cost less than the societal value of the quality-adjusted life-years [QALYs] saved) from a health care perspective. A range of estimates exist for the discounted lifetime medical care costs and the discounted QALYs for smokers versus non-smokers [13]. In the base case, we assumed no difference in treatment costs for smokers versus non-smokers (T = 0) [14], and in sensitivity analyses we evaluated scenarios with higher costs for smokers than non-smokers converted to 2015 US dollars [15]. Sloan [16] estimated the annual smoking-related healthcare costs for a 24 year old (just over $1000 per year), and Holtgrave and colleagues [6] conservatively assumed that these costs would be incurred over 27 years, discounted at 3% per annum. For discounted QALYs saved (Q) per smoking career averted, we used a base case of 1.05 QALYs saved, and in sensitivity analyses, we used a value of 1.77 QALYs saved [13]. QALYs reflect both quality and quantity of life, and 1 QALY is equivalent to 1 year of life with perfect health saved, equivalent to 2 years of life with a 50% decrement in quality of life, etc. The QALY estimates used in the present study are derived from estimates from the National Health Interview Study on the length of time in different health states for smokers and non-smokers and assigning health-related quality of life utility scores to those health states [13]. For societal willingness to pay to save one QALY (W), we used a standard of three times per capita gross domestic product (GDP) in the United States in 2015 [17], derived from World Bank estimates [18]. While other cost-per-QALY thresholds have been recommended or used, generally ranging from $50,000 to $200,000 [19], we prefer a threshold that reflects secular changes in income and inflation over time.

## 3. Results

### 3.1. Cost of the Finishit Campaign

The total costs of the *FinishIt* campaign from 2014–2016 was $162,056,543 (Table 2). These expenditures reflect costs associated with developing, delivering, and evaluating the campaign. In 2014, there were no media, grassroots tour, or vendor costs, as the campaign was only in development. Interactive media costs in 2014 were primarily for the development and launch of new interactive media, including thetruth.com website. Interactive media costs increased from 2015 to 2016 due to an assessment of the campaign’s interactive media and further investment in digital platforms.

Evaluation costs covered the development of formative, implementation and outcome evaluation components of the campaign in 2014, as well as implementation and new evaluation components in 2015 and 2016. For communications and public relations, costs were incurred in 2014 for the development of the revamped campaign communications infrastructure, and costs increased in 2015 and 2016 with the addition of new communications staff and the implementation of the campaign.

### 3.2. Cost-Effectiveness Thresholds

In the base-case, we assume that no treatment costs are saved, and the cost of the intervention is exceeded by the value of the QALYs saved when 917 smoking careers are averted. If averting a smoking career is associated with a higher number of QALYs saved (1.77), then 544 smoking careers would need to be averted for the intervention to be cost-effective. With the upper estimate for lifetime medical care costs saved, 813 smoking careers would need to be averted. With upper estimates for both treatment cost saved and QALYs averted per smoking career averted—the most optimistic scenario—506 smoking careers would need to be averted for the *FinishIt* campaign to be cost-effective (Table 3).

### 3.3. Cost-Saving Thresholds

In the base-case scenario it is not possible to reach the cost-saving threshold as we assumed that medical care costs were the same for smokers and non-smokers (T = 0). However, if a smoking career averted (T) is associated with saving $22,553 in discounted medical care costs, then 7186 smoking careers would need to be averted for the total medical care costs saved to exceed the costs of the intervention and, thus, for the *FinishIt* intervention to be cost-saving (Table 3).

## 4. Discussion

Population-level health campaigns must adapt to rapidly evolving media environments and shifts in behavioral norms to effectively reach and influence their audience. The truth *FinishIt* campaign was designed to respond to new challenges and opportunities through targeted messaging aimed at youth and young adults, with a particular emphasis on airing segmented digital media across a variety of youth-oriented platforms. We estimate the cost of the *FinishIt* campaign, over the span of three years (2014–2016), at $162 million, which is less than half the cost of the 2000–2002 truth campaign, largely due to the efficiency and lower cost of digital media [6]. Under the assumptions of the pessimistic base-case scenario, 917 smoking careers would need to be averted for the *FinishIt* campaign to be cost-effective. When we assume that smoking is associated with increased lifetime medical care costs, 544 smoking careers would need to be averted for the campaign to be cost-effective—and 7186 to be cost-saving. These thresholds are orders of magnitude lower than the impact of the original truth campaign in the early 2000s, which averted nearly 170,000 smoking careers and saved nearly 180,000 QALYs [6]. For the *FinishIt* campaign, preliminary analyses indicate that exposure to campaign ads was associated with significantly reduced smoking intentions [22]. However, only future impact analyses will provide a determination regarding whether the *FinishIt* campaign exceeds the identified cost-saving and cost-effectiveness thresholds. 

## 5. Study Limitations

Both the cost analysis and threshold analyses are not without limitations. First, this analysis relies on internal budgetary documents, and as a result, some costs may have been misclassified. To address any possible bias regarding budget allocations and establish conservative estimates, the costs reflect several expenditures not typically included in such an analysis including expenditures associated with organizational capital improvements for Truth Initiative. Secondly, published estimates regarding medical care costs and QALYs associated with smoking vary. As a result, this analysis uses pessimistic estimates in our base-case and sensitivity analyses to characterize the impact of this variation. Nonetheless, the data reflect the best estimates for a national mass media campaign with published estimates of health care costs and life expectancy associated with smoking.

## 6. Conclusions

While national anti-tobacco public education campaigns such as *FinishIt* require substantial investment, the potential positive impact to society is substantial. Savings from medical care costs, life expectancy, and quality of life can also be substantial, particularly from the deadly toll of tobacco-related disease and death. Findings here indicate that a relatively modest number of smoking careers need to be averted for such investments to be cost-saving or cost-effective.

## Figures and Tables

**Table 1:** Direct outputs of the *FinishIt* campaign (2014–2016).

Output	Description	Number of Outputs
Advertisements produced		
Video	Television and digital ads.	14
Cinema	Ads aired in movie theaters.	2
Radio	Ads aired on Pandora, Spotify, and Soundcloud, typically 8 ads per year.	24
Banners produced	Digital display banner ads, typically 10 per year.	30
Content Integrations/Digital Partnership Ads	Custom integrated content pieces within entertainment media. Three integrations per partnership, occurring 2 times per year, with 10 partners per year.	60
Homepage Takeovers	Custom content about *Finish It* that are displayed on another publisher’s homepage (e.g., BuzzFeed). Typically 12–15 takeovers per campaign with a total of 30 takeovers per year.	90
Search	Text ads that appear on Google results pages and across the Google Network, which includes the Search Network, search partners, and the Display Network.	70
Influencer Creative Content	Videos and other creative content created and posted by social media influencers about *Finish It*.	30
Website	The truth website was rebuilt in 2014 and in 2015.	2
Articles	Copy-text and images developed by staff.	45
Quizzes	Unique quizzes that promote the truth brand and/or the current campaign.	30
Activations	Activities on the truth website that engage and increase brand awareness (e.g., petitions, thank you cards, submit an idea).	15
Social Media Network	Truth-owned social media accounts (i.e., Instagram, Twitter, Snapchat, Facebook, and YouTube).	5
Social Media Posts	Unique social media posts (e.g., gifs, images) that truth published on truth-owned social media accounts.	4254
Owned and Operated Messaging	Truth created and sent out email blasts and/or text message campaigns to engage consumers.	156
Video Games	Truth incorporated custom content for an existing console game.	1
Partnerships	Partnerships with other brands (i.e., PetCo, Vans, Tyra Banks, and Coda).	4
Events	Tours, concerts, and other events, typically 100 per year.	300

**Table 2:** Costs of the *FinishIt* campaign by fiscal year (FY) and cost category reported in US $ in year expended.

Cost Category	FY 2014	FY 2015	FY 2016	All Years	Explanation and Comments
**Marketing**	5,234,500	61,074,000	70,186,000	136,494,500	Other than personal service.*
Media	0	48,440,000	55,569,000	104,009,000	TV, radio, digital, and cinema advertising.
Creative Production	497,000	2,869,000	3,263,000	6,629,000	Production costs related to our ad agency, 72andSunny, and includes all aspects of creation, talent fees, printing, and dissemination.
Interactive	1,250,000	765,000	1,945,000	3,960,000	Digital banners, social posts, or social/homepage takeovers; developing thetruth.com website, mobile commons, and digital analytics; costs associated with social production and engagement.
Grassroots/Tour	0	2,527,000	2,125,000	4,652,000	*FinishIt* tour: gear (skatedecks, sunglasses, bags, and t-shirts) storage of trucks and gear, drivers, permits, equipment, other related costs.
Audience Research	129,500	257,000	228,000	614,500	Audience segmentation studies and communication checks (i.e., focus groups, discussion boards, partnership research studies).
Vendor Travel	0	102,000	127,000	229,000	Costs incurred for our vendors (e.g., our ad agency) to travel on behalf of *FinishIt*.
Agency Fees	3,250,000	5,991,000	6,397,000	15,638,000	
General and administrative	108,000	123,000	532,000	763,000	Operational expenses related to running *FinishIt*, including rent, utilities, and insurance. The rate for marketing general and administrative expenses increased from FY 2015 to FY 2016.
**Communications**	364,560	717,850	1,991,000	3,073,410	Other than personal service.*
**Evaluation Science and Research**	3,155,161	4,726,655	3,484,817	11,366,633	Other than personal service.*
**Truth Initiative Salaries**	3,137,000	3,644,000	4,341,000	11,122,000	
**Total cost**	11,891,221	70,162,505	80,002,817	162,056,543	

* For marketing, “other than personal service” include money spent on media, supplies, travel, research, service contracts, etc. For Communications and Evaluation Science and Research include service contracts, research, supplies, travel, equipment, and general and administrative expenses.

**Table 3:** Parameter estimates and number of smoking careers averted to reach cost-saving and cost-effectiveness estimates.

Estimate	Symbol/Formula	Value	Source/Notes
Intervention costs	C	$162,056,543	Table 1; text
Discounted lifetime treatment costs for smokers vs. non-smokers	T		
Base case		$0	[14]
Upper estimate		$22,553	[16]
Quality-adjusted life years (QALYs) saved per smoking career averted	Q		
Base case		1.05	[20]
Upper estimate		1.77	[21]
Societal willingness to pay to save 1 QALY	W	3 × $56,116	3 × U.S. per capita GDP (2015) [18]
Threshold and scenario	Formula	Smoking careers averted	
Cost-saving threshold (smoking careers averted [A] to be cost-saving)	A > C/T		
Base case		Undefined	
Upper estimate T		7186	
Cost-effectiveness threshold (smoking careers averted [A] to be cost-effective)	A > C/(T + (Q × W))		
Base case		917	
Upper estimate T		813	
Upper estimate Q		544	
Upper estimate T and upper estimate Q		506	
